# Assessment of self-reported financial toxicity among patients with nasopharyngeal carcinoma undergoing radiotherapy: A cross-sectional study in western China

**DOI:** 10.3389/fonc.2022.1011052

**Published:** 2022-10-28

**Authors:** Hua Jiang, Wenxuan Mou, Jianxia Lyu, Luxi Jiang, Ying Liu, Yu Zeng, Aiping Hu, Wei Zheng, Qinghua Jiang, Shuang Yang

**Affiliations:** ^1^ School of Medicine, University of Electronic Science and Technology of China, Chengdu, China; ^2^ School of Nursing, Chengdu Medical College, Chengdu, China; ^3^ Department of Head and Neck Radiotherapy, Sichuan Cancer Hospital & Institute, Sichuan Cancer Center, School of Medicine, University of Electronic Science and Technology of China, Chengdu, China

**Keywords:** financial toxicity, financial burden, nasopharyngeal carcinoma, psychological distress, NPC (nasopharyngeal carcinoma)

## Abstract

**Objective:**

Using the Comprehensive Score for Financial Toxicity (COST) tool to measure financial toxicity (FT) among nasopharyngeal cancer (NPC) patients in western China and investigate the association between FT and psychological distress.

**Methods:**

We conducted a cross-sectional study of survivors with NPC in a tertiary oncology hospital in China. FT was assessed using the COST (Chinese version), a validated instrument widely used both at home and abroad. The NCCN Distress Thermometer (DT) was used to measure psychological distress. A multivariate logistic regression model was built to determine factors associated with FT, and the Pearson correlation was used to assess the correlation between COST and DT scores.

**Results:**

Of 210 patients included in this study, the mean FT score was 16.3 (median: 22.5, SD: 9.7), and the prevalence of FT was 66.2% (mild FT: 37.1%, moderate FT: 50.5%, severe FT: 2.4%). Suggested by the logistic regression model, 5 variables were associated with increased FT: unemployed, no commercial insurance, receiving lower annual income, advanced cancer, and receiving targeted therapy. The Pearson correlation showed a significantly moderate correlation between financial toxicity and psychological distress (r= -0.587, P < 0.001).

**Conclusion:**

Patients with nasopharyngeal carcinoma (NPC) in western China demonstrated higher self-reported financial toxicity (FT) associated with factors including unemployed, no commercial insurance, receiving lower annual income, advanced cancer, and receiving targeted therapy. These predictors will help clinicians identify potential patients with FT in advance and conduct effective psychological interventions.

## Introduction

Nasopharyngeal carcinoma (NPC) is epithelial cancer originating from the nasal mucosa lining. According to the American Cancer Society in 2018, the year saw 129,079 new cases of nasopharyngeal cancer across the world, accounting for 0.7% of the total incidence of cancer, and 72,987 deaths, 8% of the annual death toll (1, 2). China has the highest occurrence of nasopharyngeal cancer in the world, mainly in its southern and western regions (2), which accounted for 38.29% of the global incidence of nasopharyngeal cancer, its incidence (1.9/100,000) and mortality (1.2/100,000) significantly higher than the world average (1.2/100,000, 0.7/100,000) (2, 3).

Intensity-modulated radiotherapy (IMRT) and systemic chemotherapy are major treatments for patients with advanced NPC (4). Due to the occult nature of NPC in its early stages, 70% of the patients are locally advanced at the time of initial diagnosis (5). Long-term concurrent chemoradiotherapy will not only cost huge medical expenses, but also induce significant psychological distress due to side effects during the treatment, such as radiation mucositis and dysphagia. Hence, advanced NPC patients are faced with considerable financial and mental burden resulting from medical service utilization.

Financial toxicity (FT) was defined as the objective financial burden and subjective financial distress of cancer patients due to treatments using innovative drugs and concomitant health services, similar to side effects such as nausea and vomiting (6, 7). FT can be influenced by demographics, economic status, disease, treatment, etc. Taking into account differences in cultural background and health systems, the influencing factors of FT may vary among countries (8). However, current researches on FT were mainly conducted in developed countries (such as the United States) where the incidence of nasopharyngeal cancer was relatively low, causing a gap in the FT-related studies of this specific disease.

Previous studies (9–11) have shown that patients undergoing radiotherapy have a relatively high prevalence of FT, which was linked to poorer health-related quality of life, more severe psychological distress, greater symptom burden, decreased adherence to treatment, and increased mortality risk (12). When patients are unable to afford medical costs, they turn to other financial coping mechanisms including use of savings, loans, cutting back on leisure activities, reducing expenditure on necessities of life, or working longer hours, or even non-compliance including avoidance or discontinuation of prescriptions, or deferment in medical care and follow-up visits (13). Therefore, it is necessary to better understand the risk factors of FT, which are expected to improve the interventions aimed at reducing financial distress, thus improving quality care and policy optimization.

To our knowledge, FT in nasopharyngeal carcinoma patients has not yet been studied in China. This study is aimed to examine the FT of nasopharyngeal carcinoma patients, as well as the link between FT and psychological distress using the Comprehensive Score for Financial Toxicity (COST), developed and validated by De Souza et al (14). Our results are expected to assist clinicians in the quick identification of high-risk groups in patients with nasopharyngeal carcinoma by inferring the risk factors of FT concluded from this study.

## Materials and methods

### Study design and sampling plan

We conducted a survey-based cross-sectional study in three of the Head and Neck Radiotherapy departments in China between October 2021 and July 2022. All three departments are affiliated with a tertiary oncology hospital (Sichuan Cancer Hospital & Research Institute), the largest oncology hospital in Southwestern China, which guaranteed a sufficient sample size. All NPC patients were treated with image-guided radiation therapy (IGRT), usually five times a week. NPC patients are most likely to develop acute toxic side effects after 2 ~3 weeks of radiotherapy, and most patients begin to experience financial distress.

Patients were enrolled if they were (i) elder than 18 years, (ii) pathologically diagnosed with stage 0-IV (AJCC, 8^th^ edition) nasopharyngeal carcinoma, including those with recurrence and metastasis (iii) treated with radiotherapy for more than 2 weeks (as either stand-alone or part of the multimodal treatment regimen), and (iv) willing to accept this interview. The exclusion criteria contained: (i) currently treated for another malignancy, (ii) participating in other clinical trials, and (iii) unable to read, understand and speak Chinese. All patients had voluntarily signed an informed consent form before the investigation. Ethics approval was acquired from the Ethics Committee of Sichuan Cancer Hospital (SCCHEC-02-2020-067).

The sample size was determined based on the acceptable width of 95% confidence interval (CI) of FT. Assume that the sample proportion is 0.78 which was reported in a systematic review of FT in cancer survivors in China (15). To produce a confidence interval with a width of no more than 0.12, 184 subjects were needed.

### Questionnaire, variables, and outcomes

The general information questionnaire was designed based on a meta-analysis of an extensive study which involved sociodemographic and socioeconomic data. Information on the clinic data of the patients was extracted from electronic medical records from the Hospital Information System (HIS).

FT was assessed using the COST (Chinese version) instrument, validated and widely used internationally and in China (16–19). The total score ranges from 0 to 44, with lower scores indicating more severe FT in patients. According to the FT grading system established by De Souza et al., a COST score > 26 indicates no FT (grade 0), 14-25 mild FT (grade 1), 1-13 moderate FT (grade 2), and 0 suggests severe FT (grade 3) (20). The Cronbach’s α of the Chinese version of COST is 0.891.

The NCCN Distress Thermometer (DT) was used to measure psychological distress. The total score ranges from 0 (no distress) to 10 (great distress); A score of 4 has been determined to be the cut-off score for moderate psychological distress and the trigger for psychological assistance referral (21).

### Statistical analysis

Descriptive statistics were used to characterize the study population, and the prevalence of FT and its 95%CI will be reported. We compared demographic and disease characteristics among different FT groups using χ2 or Fisher’s exact tests for categorical variables, and Wilcoxon rank sum test for continuous variables. A multivariate logistic regression model was built to determine factors associated with FT (COST<26). Multivariable regression analysis included significant covariates identified in univariate analysis (*P <*0.05) and covariates thought to be of clinical significance (sex, age and immunotherapy).

Pearson correlation method was used to assess the correlation between COST and DT scores. A coefficient (r) between 0.20 and 0.39 suggested a mild correlation, 0.4-0.59 a moderate correlation, 0.60-0.79 a strong correlation, and ≥0.80 a very strong correlation (22). P<0.05 (two-tailed) was considered statistically significant. All statistical analyses were performed with SPSS 26.0 (IBM, NY, USA).

## Results

### Patient participation and characteristics

Between October 2021 and July 2022, there were 246 patients diagnosed with nasopharyngeal carcinoma who had been treated with at least 2 weeks of radiotherapy. Of these patients, 235 met our inclusion criteria; ultimately, 210 of them agreed and completed the questionnaire, with the response rate being 89.4%. The mean age of the 210 patients enrolled in our study was 51 years, ranging from 22–78 years. 34.3% of the patients were covered by Urban Basic Medical Insurance (UBMI), 49% by New Rural Cooperative Medical Insurance, while only 14.7% had private insurance. 77.1% of them had advanced nasopharyngeal carcinoma. 72.3% were receiving chemotherapy (chemotherapy regimens were paclitaxel plus cisplatin and capecitabine or gemcitabine plus cisplatin), 36.2% were receiving molecular targeted therapy (Nimotuzumab), and 23.3% were undergoing at least one immunotherapy (Carrelizumab or Toripalimab). The demographic and medical characteristics of the patients are shown in **Table 1**.

**Table 1:** Patients characteristics by COST score (*N*=210).

Medical characteristics	N	COST≥26 (n=71)	COST<26 (n=139)	*p*
Age (years) ± SD		50.02 ± 8.75	51.10 ± 10.54	0.531
Sex				0.049
Male	141	55 (39.0%)	86 (61.0%)	
Female	69	16 (23.2%)	53 (76.80%)	
Age (years)				0.126
≥65	161	46 (31.1%)	115 (68.9%)	
<65	49	21 (42.9%)	28 (57.1%)	
Place of residence				<0.001
Urban	90	43 (55.7%)	47 (44.3)	
Rural	120	28 (23.3%)	92 (96.7%)	
Marital status				0.032
Married	162	60 (37.0%)	102 (63.0%)	
Unmarried	30	6 (20.0%)	24 (35.7%)	
Divorced	12	4 (33.3%)	8 (66.7%)	
Widowed	6	3 (50.00%)	3 (50.00%)	
Education level				<0.001
Primary school (<6 year)	41	7 (17.1%)	34 (82.9%)	
High school (6~9 year)	89	23 (25.8%)	66 (74.1%)	
Vocational college (9~12 year)	49	22 (44.9%)	27 (55.1%)	
College or above (>12 year)	31	19 (61.3%)	12 (38.7%)	
Employment status				<0.001
Employed	72	34 (47.2%)	38 (52.8%)	
Unemployed	115	25 (21.7%)	90 (78.3%)	
Retired	23	15 (65.2%)	8 (34.8%)	
Health insurance				0.008
UEBMI^a^	70	25 (35.7%)	45 (64.3%)	
URBMI^b^	26	8 (30.8%)	18 (69.2%)	
NRCMI^c^	93	24 (25.8%)	69 (74.2%)	
Commercial insurance	21	14 (66.7%)	7 (33.3%)	
Annual household income (CNY)				
<60,000	75	12 (16.0%)	63 (84.0%)	<0.001
60,000~120,000	63	21 (33.3%)	42 (66.7%)	
12,000~200,000	55	26 (47.3%)	29 (52.7%)	
>200,000	17	12 (70.6%)	5 (29.4%)	
Travelling time to hospital				0.236
<30min	25	14 (56.0%)	11 (44.0%)	
30min~1h	26	15 (57.7%)	11 (42.3%)	
1~2h	67	25 (37.3%)	42 (62.7%)	
2~5h	75	12 (16.0%)	63 (84.0%)	
>5h	17	5 (29.4%)	12 (70.6%)	
Smoking				0.253
Yes	143	52 (36.4%)	91 (63.6%)	
No	67	19 (28.4%)	48 (71.6%)	
Chronic disease				0.845
Yes	37	12 (32.4%)	25 (67.6%)	
No	173	59 (34.1%)	114 (65.9%)	
Tumor stage				<0.001
**I~II**	48	30 (62.5%)	18(37.5%)	
**III~IV**	162	41 (25.3%)	121 (74.7%)	
Diagnosis time				0.173
<1month	20	5 (25.0%)	15 (75.0%)	
1~6month	144	52 (36.1%)	92 (63.9)	
6~12 month	25	8 (32.0%)	17 (68.0%)	
1~5year	19	4 (21.0%)	15 (79.00%)	
>5year	2	1 (50.0%)	1 (50.0%)	
Chemotherapy				<0.001
Yes	152	40 (26.3%)	112 (73.7%)	
No	58	31 (53.4%)	27 (46.6%)	
Immunity therapy				0.219
Yes	49	13 (26.5%)	46 (73.5%)	
No	161	58 (36.0%)	103 (64.0%)	
Targeted therapy				<0.001
Yes	76	10 (13.1%)	66 (86.9%)	
No	134	61 (45.5%)	73 (54.5%)	

UEBMI ^a^, The Urban Employees’ Basic Medical Insurance Scheme covering 70–90% of the medical expenses; URBMI ^b^, The Urban Residents’ Basic Medical Insurance Scheme covering 50–70% of the medical expenses; NCMS ^c^, The New Cooperative Medical Scheme covering 50–60% of the medical expenses; CNY, Chinese Yuan.

Travelling time to hospital: The travel time that one patient spends on journal from home to hospital.

### Financial toxicity

The mean FT score was 16.3 (median 22.5, SD 9.7). 35.7% of the included patients had an annual household income below 60,000 CNY, 30.0% between 60,000 and 120,000 CNY, and 26.2% between 120,000 and 200,000 CNY (1CNY=0.15USD, as of 2022/7/20). The prevalence of FT was 66.2% (95CI: 59.7~72.6), among which 37.1% reported mild FT, 50.5% moderate FT, and 2.4% severe FT. The distribution of FT severity is listed in **Table 2**.

**Table 2:** Distribution of financial toxicity in patients with nasopharyngeal carcinoma (N=210).

Grading	No. of patients	Proportion (95%*CI, %*)
No FT (Grade0)	71	33.8 (95%*CI*:27.4~40.3)
Mild FT (Grade1)	78	37.1 (95%*CI*:30.6~43.7)
Moderate FT Grade2)	106	50.5 (95%*CI*:43.7~57.3)
Severe FT (Grade3)	5	2.4 (95%*CI*:0.3~4.5)

### Variables associated with financial toxicity

**Figure 1:**
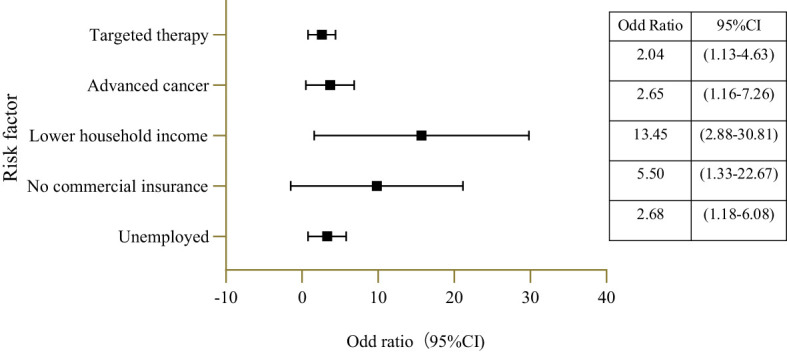
Forest plot of risk factors for self-reported financial toxicity in NPC patients.

The univariate analysis of baseline variables associated with FT was described in **Table 3**. As shown by the analysis, patients reporting FT tended to be younger, living in rural areas, unmarried, of lower educational level, unemployed, no private insurance, receiving lower annual income, receiving immunity therapy and receiving targeted therapy (**Table 3**). After adjusting for potentially confounding variables in the multivariable modeling, we found the following factors associated with increased financial toxicity: unemployed, advanced cancer, no private insurance, lower income, and receiving targeted therapy (**Figure 1**). Compared to annual household income > 200,000 CNY, patients with annual household income below 60,000 CNY had higher odds of reporting FT (odd ratio [OR], 13.45; p<0.001). Compared with commercial insurance, patients who rely only on NRCMI (odd ratio [OR], 5.50; p=0.018) and URBMI (odd ratio [OR], 5.40; p=0.017) had higher odds of reporting FT.

**Table 3:** Univariable and multivariable logistic regression models predicting the likelihood of self-reported financial toxicity.

Characteristics	Univariate analysis		Multivariable analysis	
	*OR* (95% *CI*)	*P*-value	*OR* (95% *CI*)	*P*-value
Age (years)				
≥65	Reference		Reference	
<65	1.66 (0.86-3.21)	0.051	1.91 (0.70-8.19)	0.205
Sex			
Male	Reference		Reference	
Female	1.90 (1.02-3.62)	0.128	1.44 (0.63-3.30)	0.382
Place of residence				
Urban	Reference		Reference	
Rural	3.01 (1.66-5.42)	< 0.001	1.33 (0.58-3.01)	0.493
Marital status				
Married	Reference		Reference	
Unmarried	2.35 (1.21-6.08)	0.027	2.45 (0.77-8.20)	0.143
Divorced	1.17 (0.73-4.57)	0.789	3.80 (0.12-15.35)	0.440
Widowed	2.94 (0.33-10.64)	0.330	2.77 (0.22-34.50)	0.427
Education level				
College or above (>12 year)	Reference		Reference	
Primary school (<6 year)	3.76 (1.38-10.27)	0.010	3.14 (0.81-12.22)	0.099
High school (6~9 year)	4.19 (1.76-9.94)	0.002	2.88 (0.89-9.31)	0.077
Vocational college (9~12 year)	1.26 (0.51-3.12)	0.155	1.27(0.37-4.35)	0.693
Employment status				
Employed	Reference		Reference	
Unemployed	3.22 (1.69-6.11)	< 0.001	2.68 (1.18-6.08)	**0.018**
Retired	0.82 (0.32-2.10)	0.679	0.76 (0.22-2.66)	0.673
Health insurance				
Commercial insurance	Reference		Reference	
UEBMI	3.60 (1.28-10.09)	0.017	4.52(0.91-22.35)	0.064
URBMI	4.50 (1.31-15.42)	0.001	5.40 (1.37-21.50)	**0.017**
NRCMI	5.75 (2.07-15.93)	0.015	5.50 (1.33-22.67)	**0.018**
Annual household income (CNY)				
>200,000	Reference		Reference	Reference
<60,000	12.60 (3.75-42.34)	< 0.001	13.45 (2.88-30.81)	**0.001**
60,000~120,000	4.80 (1.49-15.42)	0.008	5.57 (1.28-24.18)	**0.022**
12,000~200,000	2.67 (0.83-8.62)	0.099	5.76 (1.25-26.57)	**0.025**
Tumor stage				
**I~II**	Reference		Reference	
**III~IV**	4.92 (2.48-9.74)	< 0.001	2.65 (1.16-7.26)	**0.030**
Chemotherapy				
No	Reference		Reference	
Yes	3.21 (1.73-6.03)	< 0.001	1.77 (0.67-4.63)	0.152
Immunity therapy				
No	Reference		Reference	
Yes	1.59 (0.76-3.17)	0.221	1.10 (0.44-2.73)	0.152
Targeted therapy				
No	Reference		Reference	
Yes	3.13 (1.65-5.92)	< 0.001	2.04 (1.13-4.63)	**0.042**

The bold values means p<0.05.

### Financial toxicity and psychological distress

**Figure 2:**
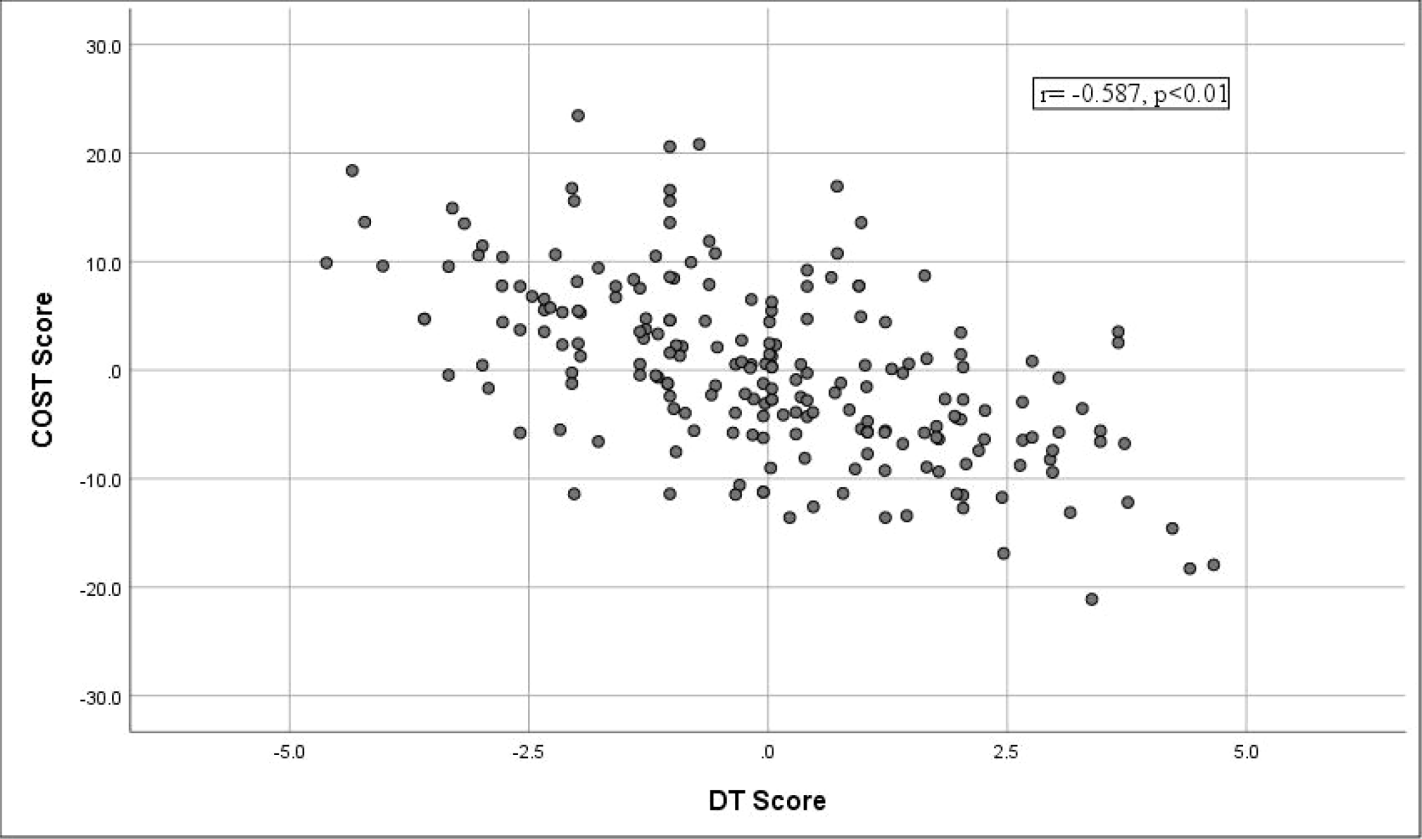
Correlation between financial toxicity and psychological distress.

The mean DT score in the overall study population was 4.72 (SD=2.07). The Pearson correlation coefficient (r) between the COST score and DT score was -0.65 (P < 0.001). After adjusting for covariates such as age, sex, marital status and treatment, the correlation coefficient between COST and DT was -0.587, representing a moderate correlation between FT and psychological distress, as demonstrated in **Figure 2**, where FT increased (lower COST score) with psychological distress (higher DT score).

## Discussion

Our study found that FT was highly prevalent among NPC patients, with a prevalence of 66.2%, mostly reporting moderate FT, although health insurance coverage varied from patient to patient. Several studies (23–25) have shown that FT was common among Head and Neck Cancer (HNC) survivors, and the mean COST scores in our study were even lower than indicated by previous results, implying greater financial-related risk that patients in this study were suffering from. On the one hand, nasopharyngeal carcinoma patients were mainly males with the age of onset concentrated between 40 and 59 years (2). In China, people of this age, especially men, are often burdened with enormous living pressures coupled with the duties of supporting both children and the elder (26). On the other hand, the incidence of nasopharyngeal carcinoma has prominent regional characteristics, with most of the patients investigated in this study coming from China’s western region. This is probably due to the fact that the economy of western China is less developed, thus resulting in lower overall income level compared with the east.

Our findings in this study suggested that unemployed, no commercial insurance, receiving lower annual income, advanced cancer, and receiving targeted therapy were factors significantly associated with higher FT. Lower annual household income is one of the key risk factors of FT. We observed a substantial decrease in the probability of reporting FT in patients with annual household income above 200,000 CNY, which was consistent with the results of Xu and Jing et al. that investigated FT in lung cancer and breast cancer patients respectively (27, 28). The results of Yu et al. found that patients with URBMI were associated with an average 2.2 point decrease in the COST scores compared to patients who had UEBMI (29). In this study, we found that patients without commercial insurance are at greater risk of suffering from FT, whether the patient had URBMI or UEBMI. Universal health insurance has been developed in China, but the current tiered “basic medical insurance” scheme cannot cover all the health services. Social medical insurance in China contains three types which are UEMBI for urban employees, URMBI for urban residents, and NRCMI for rural residents, but their reimbursement coverage was limited compared with commercial insurance (30). Besides, NPC has a significant impact on the work of patients after treatment, and many patients are at risk of incapacity or unemployment. Alison and Mols et al. (31, 32)confirmed that unemployment was significantly associated with FT and that those with limited financial resources were most at risk. Regarding disease characteristics, advanced cancer and receiving targeted therapy are both risk factors for FT. Advanced nasopharyngeal cancer requires more systemic treatments, such as innovative drugs and diagnostic methods, thereby directly increasing the medical costs (33). Nevertheless, several studies (11, 34, 35) have suggested that indirect medical costs for cancer treatment, such as transportation, accommodation, and time expenditure can also contribute to the FT of cancer survivors, but we did not observe significant association between travelling distance to the hospital and FT levels. The impact of indirect costs is limited probably due to the current convenience in transportation, as well as coverage of high-speed railway and subway in the areas where we conducted our survey.

Lentz et al. (36) believed that measuring psychological distress was necessary since financial distress was more severe than physical, emotional, and spiritual suffering. Two cross-sectional survey conducted by Meeker and Margret et al. confirmed that FT in cancer patients was strongly associated with psychological distress (37, 38). In the Pearson correlation analysis, we found that FT was significantly associated with psychological distress among patients with NPC, further validating their findings. Therefore, developing effective interventions to deal with patients’ financial stress has potential value in relieving their psychological distress.

Several limitations need to be considered. Although we attempted to include as many samples as possible, the single-center nature of this study still hindered the generalizability of our conclusions, and thus more multicenter cohorts are necessary to further verify the risk factors of FT. Second, the samples in our study were all insured patients. However, considering that uninsured patients in China are supposed to be more susceptible to FT, follow-up studies need to include this group of people. Given that patients’ out-of-pocket (OOP) costs are confidential, information was hard to obtain on all direct medical expenses during treatment, so it was not possible to measure the relationship between OOP costs and FT.

This is the first study using the COST tool in the nasopharyngeal carcinoma (NPC) population, and the results showed that patients with NPC in western China reported a higher proportion of FT. This result also showed a moderate association between FT and psychological distress and that patients with lower income levels are most vulnerable to FT, so it is necessary to conduct effective psychological interventions for the these highly susceptible patients. Furthermore, we identified several factors significantly associated with FT, which will assist in rapid identification of high-risk patients, and implementation of policy-level interventions.

## Conclusion

In western China, increased self-reported financial toxicity (FT) in patients with nasopharyngeal carcinoma (NPC) was mainly associated with factors including unemployed, no commercial insurance, receiving lower annual income, advanced cancer, and receiving targeted therapy. This study demonstrated the feasibility of the COST tool in NPC patients, and also revealed a moderate correlation between FT and psychological distress. Clinicians are supposed to identify potential patients with FT by these predictors at an early stage and also conduct effective psychological interventions.

## Data availability statement

The original contributions presented in the study are included in the article/supplementary material. Further inquiries can be directed to the corresponding author.

## Ethics statement

The studies involving human participants were reviewed and approved by Human Research Ethics Committee of Sichuan Cancer Hospital, Approve Number: SCCHEC-02-2020-067. The patients/participants provided their written informed consent to participate in this study.

## Author contributions

HJ and QJ conceived the idea and contributed to the conception of the study. YL and YZ were responsible for performing the systematic literature search. WM and HJ were responsible for performing data collection and analysis. JL and AH performed the data synthesis and designed tables and Figures. WZ, LJ, SY, and HJ wrote the manuscript. All authors contributed to the article and approved the submitted version.

## Funding

The study was supported by a financial grant from the Science and technology department of Sichuan Province 2020 research project, NO. 2020YFS0411.

## Conflict of interest

The authors declare that the research was conducted in the absence of any commercial or financial relationships that could be construed as a potential conflict of interest.

## Publisher’s note

All claims expressed in this article are solely those of the authors and do not necessarily represent those of their affiliated organizations, or those of the publisher, the editors and the reviewers. Any product that may be evaluated in this article, or claim that may be made by its manufacturer, is not guaranteed or endorsed by the publisher.

## References

[1] Bray F Ferlay J Soerjomataram I Siegel RL Torre LA Jemal A. Global cancer statistics 2018: GLOBOCAN estimates of incidence and mortality worldwide for 36 cancers in 185 countries. CA Cancer J Clin (2018) 68(6):394–424. doi: 10.3322/caac.21492. [published correction appears in CA Cancer J Clin. 2020 Jul;70(4):313]. 30207593

[2] Chen YP Chan ATC Le QT Blanchard P Sun Y Ma J. Nasopharyngeal carcinoma. Lancet (2019) 394(10192):64–80. doi: 10.1016/S0140-6736(19)30956-0 31178151

[3] Xin L Jian Y Ting G Zhichao Z Yanjia C Rongtao Z. Nasopharynx cancer epidemiology in China. China Cancer (2016) 25(11):835–40. doi: 10.11735/j.issn.1004-0242.2016.11.A001

[4] Lee A Ng WT Chan J Corry J Mäkitie A Mendenhall WM. Management of locally recurrent nasopharyngeal carcinoma. Cancer Treat Rev (2019) 79:101890. doi: 10.1016/j.ctrv.2019.101890 31470314

[5] Xiao C Wang L Jiao Y Sun K Qin S Xu X. Long-term results of concurrent chemoradiotherapy for T3/T4 locally advanced nasopharyngeal carcinoma. Mol Clin Oncol (2013) 1(3):507–10. doi: 10.3892/mco.2013.75 PMC3916170 24649201

[6] Zafar SY Abernethy AP. Financial toxicity, part I: a new name for a growing problem. Oncol (Williston Park N.Y.) (2013) 27(2):80–149. PMC4523887 23530397

[7] Zafar SY Abernethy AP. Financial toxicity, part II: how can we help with the burden of treatment-related costs? Oncol (Williston Park N.Y.) (2013) 27(4):253–6. 23781687

[8] Smith GL Lopez-Olivo MA Advani PG Ning MS Geng Y Giordano SH. Financial burdens of cancer treatment: A systematic review of risk factors and outcomes. J Natl Compr Canc Netw (2019) 17(10):1184–92. doi: 10.6004/jnccn.2019.7305 PMC7370695 31590147

[9] Fabian A Domschikowski J Greiner W Bockelmann G Karsten E Rühle A. Financial toxicity in cancer patients treated with radiotherapy in Germany-a cross-sectional study. Strahlenther Onkol (2022) 27(8):299–301. doi: 10.1007/s00066-022-01936-z PMC9700565 35467099

[10] Palmer JD Patel TT Eldredge-Hindy H Keith SW Patel T Malatesta T. Patients undergoing radiation therapy are at risk of financial toxicity: A patient-based prospective survey study. Int J Radiat Oncol Biol Phys (2018) 101(2):299–305. doi: 10.1016/j.ijrobp.2018.03.014 29726359

[11] D’Rummo KA Miller L TenNapel MJ Shen X. Assessing the financial toxicity of radiation oncology patients using the validated comprehensive score for financial toxicity as a patient-reported outcome. Pract Radiat Oncol (2020) 10(5):e322–9. doi: 10.1016/j.prro.2019.10.005 31634632

[12] Carrera PM Kantarjian HM Blinder VS. The financial burden and distress of patients with cancer: Understanding and stepping-up action on the financial toxicity of cancer treatment. CA Cancer J Clin (2018) 68(2):153–65. doi: 10.3322/caac.21443 PMC6652174 29338071

[13] Zafar SY. Financial toxicity of cancer care: It’s time to intervene. J Natl Cancer Inst (2015) 108(5):djv370. doi: 10.1093/jnci/djv370 26657334

[14] de Souza JA Yap BJ Hlubocky FJ Wroblewski K Ratain MJ Cella D. The development of a financial toxicity patient-reported outcome in cancer: The COST measure. Cancer (2014) 120(20):3245–53. doi: 10.1002/cncr.28814 24954526

[15] Xu B Hu L Cheng Q So WKW. A systematic review of financial toxicity among cancer patients in China. Asia Pac J Oncol Nurs (2022) 9(8):100071. doi: 10.1016/j.apjon.2022.04.010 35692729 PMC9184292

[16] Ripamonti CI Chiesi F Di Pede P Guglielmo M Toffolatti L Gangeri L. The validation of the Italian version of the COmprehensive score for financial toxicity (COST). Support Care Canc (2020) 28(9):4477–85. doi: 10.1007/s00520-019-05286-y 31925533

[17] Honda K Gyawali B Ando M Kumanishi R Kato K Sugiyama K. Prospective survey of financial toxicity measured by the comprehensive score for financial toxicity in Japanese patients with cancer. J Glob Oncol (2019) 5:1–8. doi: 10.1200/JGO.19.00003 PMC6550026 31070981

[18] Durber K Halkett GK McMullen M Nowak AK. Measuring financial toxicity in Australian cancer patients - validation of the COmprehensive score for financial toxicity (FACT COST) measuring financial toxicity in Australian cancer patients. Asia Pac J Clin Oncol (2021) 17(4):377–87. doi: 10.1111/ajco.13508 33567158

[19] Yu H Bi X Liu Y. Reliability and validity of the Chinese version on comperhensive scores for financial toxicity based on the patient-reported outcome measures. Chin J Epidemiol (2017) 38(8):1118–20. doi: 10.3760/cma.j.issn.0254-6450.2017.08.024 28847066

[20] De Souza JA Wroblewski K Proussaloglou E Nicholson L Hantel A Wang Y. Validation of a financial toxicity (FT) grading system. J Clin Oncol (2017) 35(15_suppl):6615–5. doi: 10.1200/JCO.2017.35.15_suppl.6615

[21] Holland JC Bultz BD National comprehensive Cancer Network (NCCN). The NCCN guideline for distress management: A case for making distress the sixth vital sign. . J Natl Compr Canc Netw (2007) 5(1):3–7. 17323529

[22] Borenstein M Hedges LV Higgins JPT Rothstein HR. (2009).

[23] Beeler WH Bellile EL Casper KA Jaworski E Burger NJ Malloy KM. Patient-reported financial toxicity and adverse medical consequences in head and neck cancer. Oral Oncol (2020) 101:104521. doi: 10.1016/j.oraloncology.2019.104521 31877502 PMC7008081

[24] Mady LJ Lyu L Owoc MS Peddada SD Thomas TH Sabik LM. Understanding financial toxicity in head and neck cancer survivors. Oral Oncol (2019) 95:187–93. doi: 10.1016/j.oraloncology.2019.06.023 31345389

[25] Lu L O’Sullivan E Sharp L. Cancer-related financial hardship among head and neck cancer survivors: Risk factors and associations with health-related quality of life. Psychooncology (2019) 28(4):863–71. doi: 10.1002/pon.5034 30779397

[26] Su M Lao J Zhang N Wang J Anderson RT Sun X. Financial hardship in Chinese cancer survivors. Cancer (2020) 126(14):3312–21. doi: 10.1002/cncr.32943 32396242

[27] Xu T Xu L Xi H Zhang Y Zhou Y Chang N. Assessment of financial toxicity among patients with advanced lung cancer in Western China. Front Public Health (2022) 9:754199. doi: 10.3389/fpubh.2021.754199 35096733 PMC8790143

[28] Jing J Feng R Zhang X Li M Gao J. Financial toxicity and its associated patient and cancer factors among women with breast cancer: A single-center analysis of low-middle income region in China. Breast Cancer Res Treat (2020) 181(2):435–43. doi: 10.1007/s10549-020-05632-3 32306169

[29] Yu HH Yu ZF Li H Zhao H Sun JM Liu YY. The COmprehensive score for financial toxicity in China: Validation and responsiveness. J Pain Symptom Manage (2021) 61(6):1297–1304.e1. doi: 10.1016/j.jpainsymman.2020.12.021 33412268

[30] Mao W Tang S Zhu Y Xie Z Chen W. Financial burden of healthcare for cancer patients with social medical insurance: A multi-centered study in urban China. Int J Equity Health (2017) 16(1):180. doi: 10.1186/s12939-017-0675-y 29017542 PMC5635570

[31] Pearce A Tomalin B Kaambwa B Horevoorts N Duijts S Mols F. Financial toxicity is more than costs of care: the relationship between employment and financial toxicity in long-term cancer survivors. J Cancer Surviv (2019) 13(1):10–20. doi: 10.1007/s11764-018-0723-7 30357537

[32] Mols F Tomalin B Pearce A Kaambwa B Koczwara B. Financial toxicity and employment status in cancer survivors. a systematic literature review. Support Care Canc (2020) 28(12):5693–708. doi: 10.1007/s00520-020-05719-z PMC7686183 32865673

[33] Hui EP Ma BBY Chan ATC. The emerging data on choice of optimal therapy for locally advanced nasopharyngeal carcinoma. Curr Opin Oncol (2020) 32(3):187–95. doi: 10.1097/CCO.0000000000000622 32175925

[34] Mejri N Berrazega Y Boujnah R Rachdi H El Benna H Labidi S. Assessing the financial toxicity in Tunisian cancer patients using the comprehensive score for financial toxicity (COST). Support Care Canc (2021) 29(7):4105–11. doi: 10.1007/s00520-020-05944-6 33404807

[35] Huey RW George GC Phillips P White R Fu S Janku F. Patient-reported out-of-Pocket costs and financial toxicity during early-phase oncology clinical trials. Oncologist (2021) 26(7):588–96. doi: 10.1002/onco.13767 PMC8265355 33783054

[36] Lentz R Benson AB 3rd Kircher S. Financial toxicity in cancer care: Prevalence, causes, consequences, and reduction strategies. J Surg Oncol (2019) 120(1):85–92. doi: 10.1002/jso.25374 30650186

[37] Meeker CR Wong YN Egleston BL Hall MJ Plimack ER Martin LP. Distress and financial distress in adults with cancer: An age-based analysis. J Natl Compr Canc Netw (2017) 15(10):1224–33. doi: 10.6004/jnccn.2017.0161 PMC7569506 28982748

[38] Rosenzweig M West M Matthews J Stokan M Yoojin Kook YK Gallups S. Financial toxicity among women with metastatic breast cancer. Oncol Nurs Forum (2019) 46(1):83–91. doi: 10.1188/19.ONF.83-91 30547962

